# Supplementary material for: Quantifying the relative importance of genetics and environment on the comorbidity between mental and cardiometabolic disorders using 17 million Scandinavians
Source: Nat Commun. 2024 Jun 13;15:5064. doi: 10.1038/s41467-024-49507-3 (PMC11176385; doi:10.1038/s41467-024-49507-3)
Supplement: Supplementary file 1 — Supplementary Information [file 41467_2024_49507_MOESM1_ESM.pdf]

Supplementary Figures

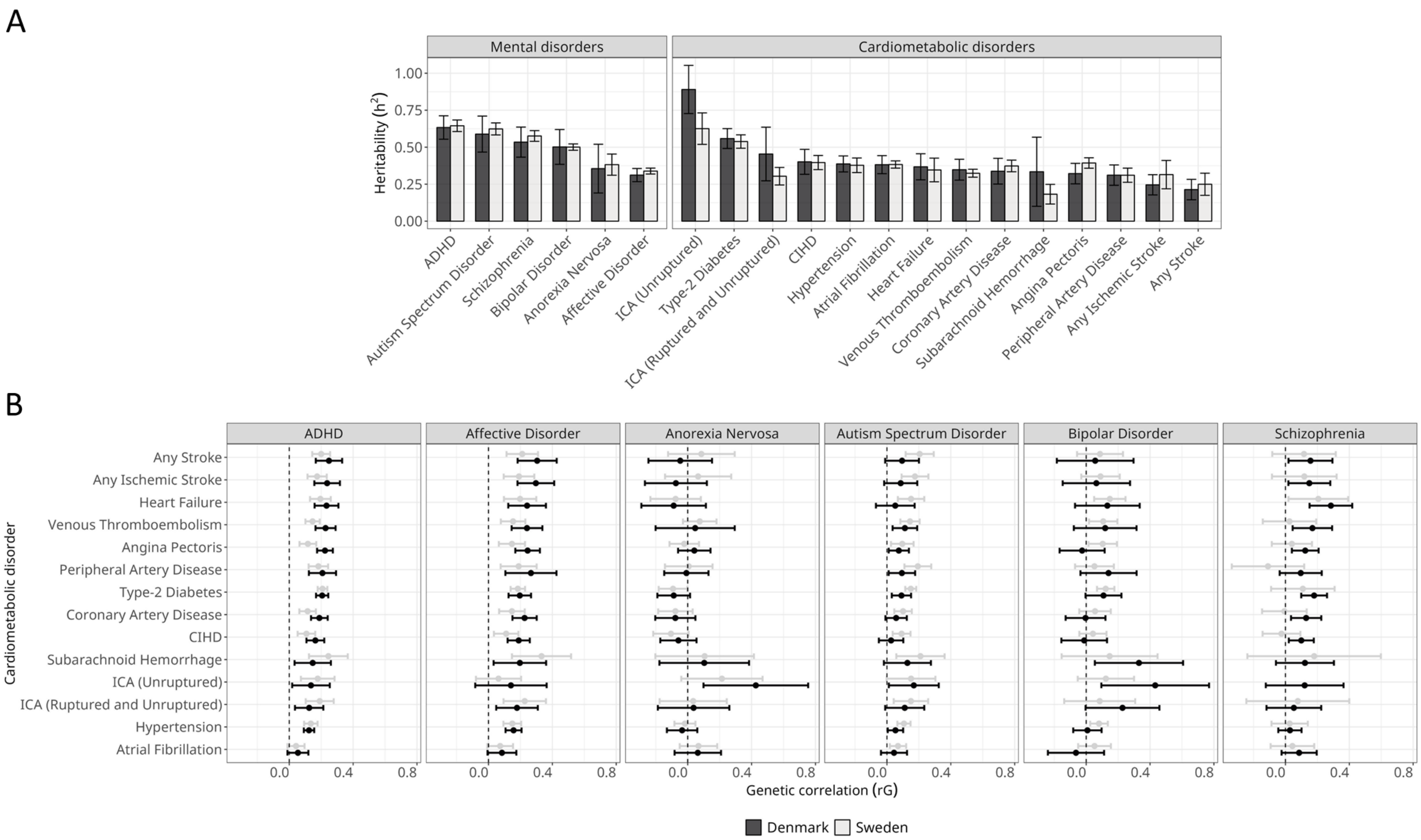

Supplementary figure S1: Comparison of Danish and Swedish register-based estimates and 95% confidence intervals for A.) heritability (h^2) B.) genetic correlations (r_g). *ADHD = attention deficit/hyperactivity disorder, ICA = intracranial aneurysm, CIHD = chronic ischemic heart disease. Heritability estimates were calculated using the full available records of Denmark (n=7,797,622) and Sweden (n= 13,222,453). Genetic correlations were estimated using Danish (n=1,560,901) and Swedish (n=2,566,100) individuals born between 1981 and 2005 followed to 2012*

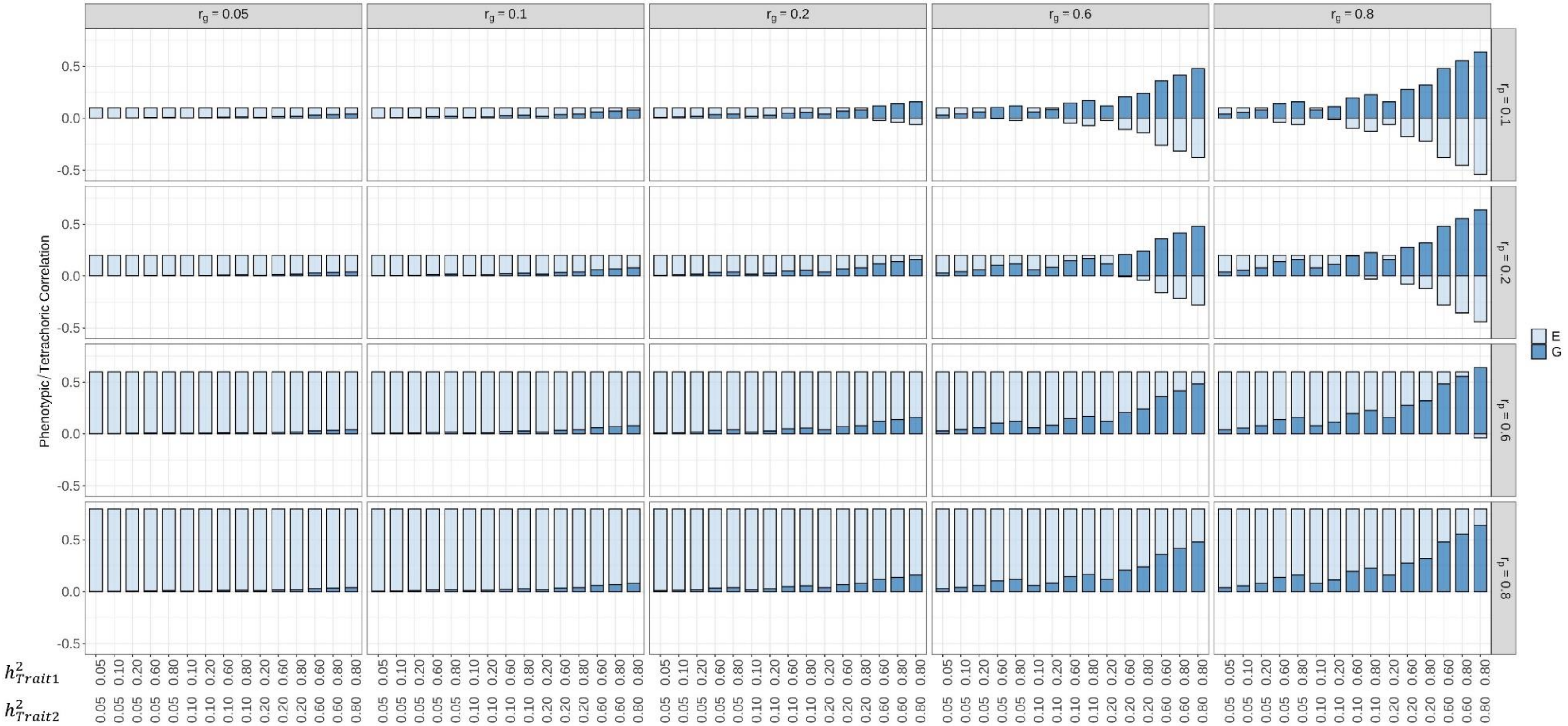

Supplementary figure S2: The effect of heritability (h^2) and genetic correlation (r_g) size on the magnitude of the genetic (G) and non-genetic (E) contribution to the phenotypic correlation (r_p) between two traits. *Phenotypic correlations (r_p) were derived using simulated hazard ratios (HZ) and prevalence's (Pr) of: r_p = 0.1: HZ=1.28, Pr=0.05; r_p=0.2: HZ=1.61, Pr=0.12; r_p=0.6: HZ=4, Pr=0.2; r_p=0.8: HZ=4, Pr=0.5.*

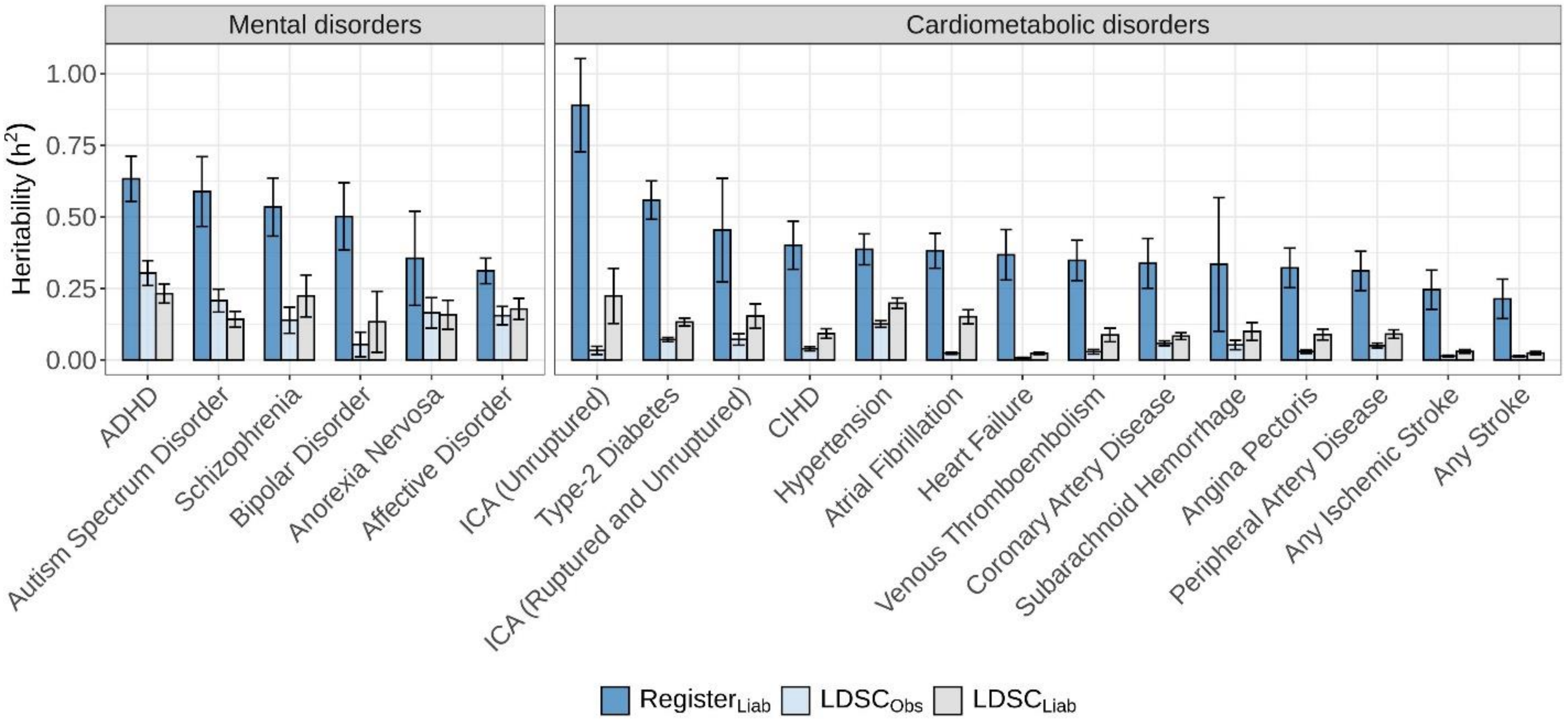

Supplementary figure S3: Narrow sense heritability (h^2) estimates and 95% confidence intervals of mental- and cardiometabolic disorders using the Danish registers ($Register_{Liab}$) and LD-score regression on the observed ($LDSC_{Obs}$) and liability scale ($LDSC_{Liab}$). *ADHD = attention deficit/hyperactivity disorder, ICA = intracranial aneurysm, CIHD = chronic ischemic heart disease.*

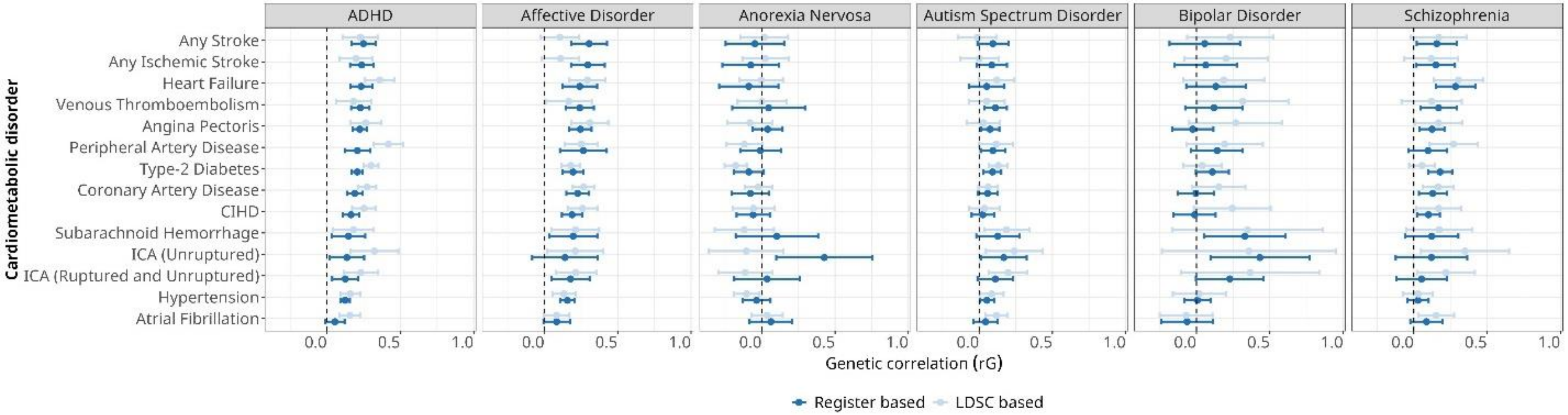

Supplementary figure S4: Comparison of the genetic correlations and 95% confidence intervals between mental- and cardiometabolic disorders calculated using register data (r_g) and LDSC ($r_{g\ SNP}$). *ADHD = attention deficit/hyperactivity disorder, ICA = intracranial aneurysm, CIHD = chronic ischemic heart disease. Register based genetic correlations were estimated using Danish (n=1,560,901) and Swedish (n=2,566,100) individuals born between 1981 and 2005 followed to 2012*

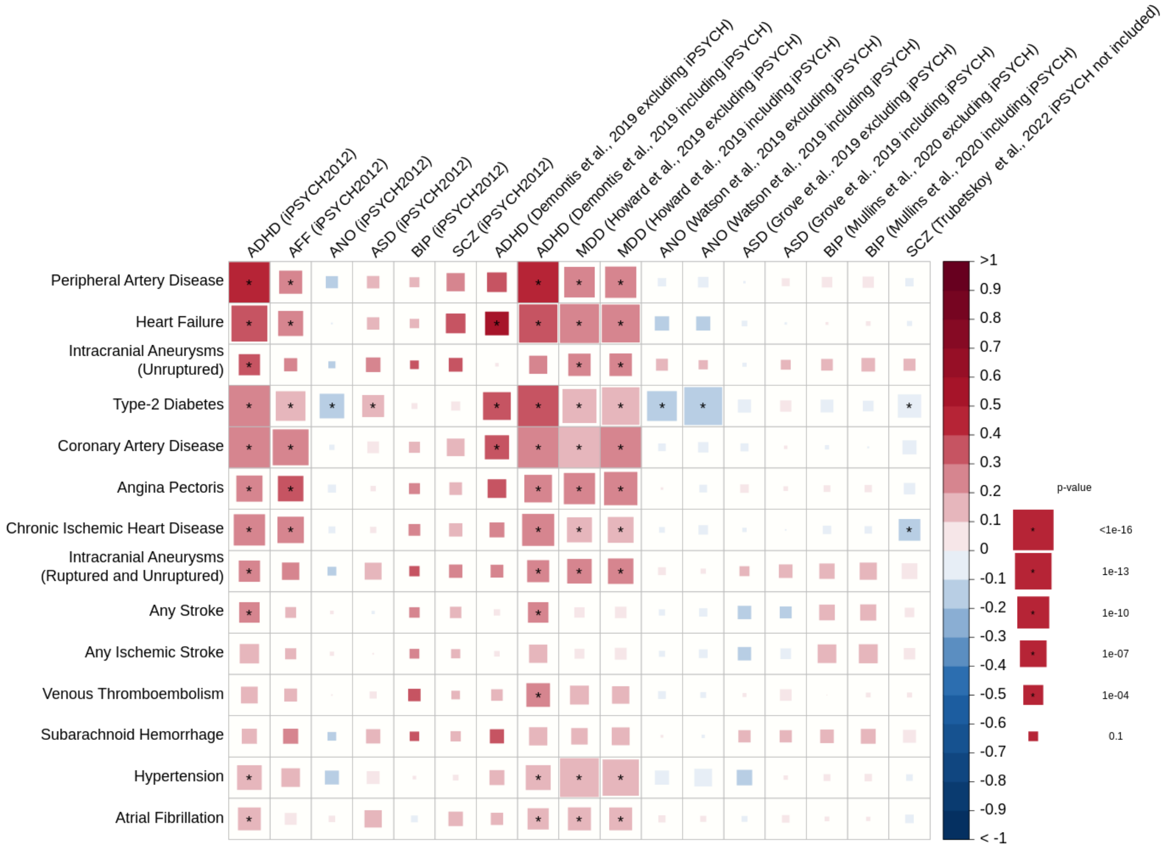

Supplementary figure S5: LDSC genetic correlations between six mental- and 14 cardiometabolic disorders. Genetic correlations ($r_{g\ SNP}$) with standard error, and p-values. *We repeated the analysis three times: using only iPSYCH samples and using the Psychiatric Genomics Consortium (PGC) meta-analysis including and excluding iPSYCH samples. Trubetskoy et al, 2022 did not contain iPSYCH samples. No PGC-AFF disorder GWAS meta-analysis exists therefore we used MDD as the next closest disorder.*

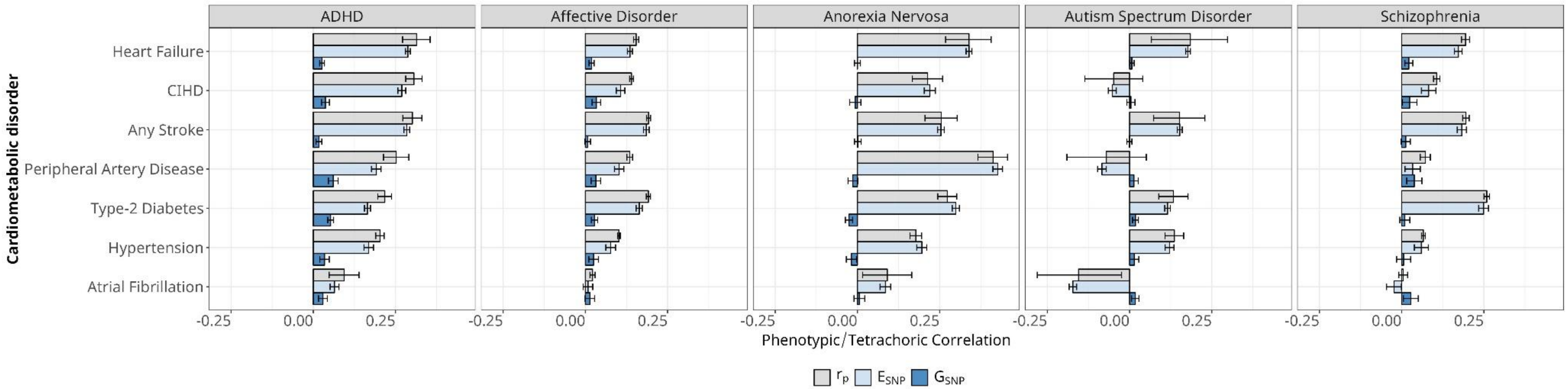

Supplementary figure S6: Quantification of the contribution of genetic (G_{SNP}) and the non-genetic (E_{SNP}) factors to the observed phenotypic correlation (r_p) **between mental- and cardiometabolic disorders using LDSC based genetic correlations ($r_{g\ SNP}$) and heritability (h^2_{SNP}) estimates.** *Estimates of r_p were selected from Momen et al.*[1]*.*
