# Supplementary material for: Quantifying the relative importance of genetics and environment on the comorbidity between mental and cardiometabolic disorders using 17 million Scandinavians
Source: Nat Commun. 2024 Jun 13;15:5064. doi: 10.1038/s41467-024-49507-3 (PMC11176385; doi:10.1038/s41467-024-49507-3)
Supplement: Supplementary file 3 — Description of Additional Supplementary Files [file 41467_2024_49507_MOESM3_ESM.pdf]

Description of Additional Supplementary Files

File Name: Supplementary Data 1A-G
Description: Cumulative incidences for a.) ADHD, b.) affective disorder, c.) anorexia, d.) autism spectrum disorder, e.) bipolar disorder, f.) schizophrenia, and g.) all cardiometabolic disorders shown for the general population and familial risk (Full-sibling and Parent-offspring) using Danish register data.

File Name: Supplementary Data 2
Description: Heritability (h^2) estimates of mental- and cardiometabolic disorders derived using register data in both Denmark and Sweden. Between country comparison of heritability estimates was performed via a two-sample two-sided Z statistic. Correlation estimates between country estimates are reported as a pearson (r) correlation coefficient. A Bonferroni significance threshold p-value (0.05/21): 2.38×10^{-3} was used to account for multiple testing.

File Name: Supplementary Data 3
Description: Heritability of disorders in the literature. ^h^2 of MDD, #Reported value derived by applying falconer's formula to observed monozygotic and dizygotic twin correlations. ADHD = attention deficit/hyperactivity disorder, ASD = Autism spectrum disorder, ANO = Anorexia nervosa, AFF = Affective disorder, BIP = Bipolar disorder, SCZ = Schizophrenia, ICA (R&U) = intracranial aneurysms (ruptured and unruptured), ICA (U) = intracranial aneurysms (unruptured), SAH = Subarachnoid hemorrhage, PAD = Peripheral artery disease, VET = Venous thromboembolism, AS = Any stroke, AIS = Any ischemic stroke, AF = Atrial fibrillation, HF = Heart failure, CAD = Coronary artery disease, T2D = Type-2 diabetes, AP = Angina pectoris, CIHD = Chronic ischemic heart disease, HYP = Hypertension

File Name: Supplementary Data 4A
Description: Genetic correlation (r_g) estimates between mental- and cardiometabolic disorders derived using register data in both Denmark and Sweden. Between country comparison of genetic correlation estimates was performed via a two-sample two-sided Z statistic. Correlation estimates between country estimates are reported as a pearson (r) correlation coefficient. A Bonferroni significance threshold p-value (0.05/90): 5.56×10^{-4} was used to account for multiple testing.

File Name: Supplementary Data 4B
Description: Genetic correlation (r_g) estimates between MDD and cardiometabolic disorders derived using register data in both Denmark and Sweden.

File Name: Supplementary Data 5
Description: Quantification of the genetic and environmental contributions to the observed comorbidity (HR) and r_p between mental and cardiometabolic disorders using heritability and genetic correlation estimates derived from register (h^2 and r_g) or genotype (h^2_{SNP} and $r_{g\text{-}SNP}$) estimates. ^Prevalence estimates by weighting the individual estimates (1981-2005 using medical records up to 2012) by the inverse of their sampling variances. *Phenotypic correlation derived using Moment et al, hazard ratios and calculated prevalence

File Name: Supplementary Data 6
Description: Summary level information on GWAS summary statistics used for PGS and LDSC estimates. ADHD = attention deficit/hyperactivity disorder, ASD = Autism spectrum disorder, ANO = Anorexia nervosa, AFF = Affective disorder, BIP = Bipolar disorder, SCZ = Schizophrenia, ICA (R&U) = intracranial aneurysms (ruptured and unruptured), ICA (U) = intracranial aneurysms (unruptured), SAH = Subarachnoid hemorrhage, PAD = Peripheral artery disease, VET = Venous thromboembolism, AS = Any stroke, AIS = Any ischemic stroke, AF = Atrial fibrillation, HF = Heart failure, CAD = Coronary artery disease, T2D = Type-2 diabetes, AP = Angina pectoris, CIHD = Chronic ischemic heart disease, HYP = Hypertension, EUR = European and EAS = East-Asian.

File Name: Supplementary Data 7
Description: Heritability (h^2_{SNP}) estimates of mental- and cardiometabolic disorders derived using LDSC on the observed and liability scale. Comparison of SNP based heritability estimates (h^2_{SNP}) and register based meta-analysis h^2 estimates was performed via a two-sample two-sided Z statistic. A Bonferroni significance threshold p-value (0.05/20): $2.5x10^{-3}$ was used to account for multiple testing. k :Population prevalence reported in Paper, l: Liability threshold derived from k, and M: Prevalence in GWAS.

File Name: Supplementary Data 8
Description: Genetic correlation (r_{g-SNP}) estimates between mental- and cardiometabolic disorders derived using LDSC. Comparison of SNP based genetic correlation estimates (r_{g-SNP}) and register based Genetic correlation estimates was performed via a two-sample two-sided Z statistic. A Bonferroni significance threshold p-value (0.05/20): $2.5x10^{-3}$ was used to account for multiple testing. *p-value < 0.05, **<0.05/14, ***<0.05/84. [2]No PGC Affective disorder GWAS exists therefore we used the PGC MDD GWAS of Howard et al., 2019 as the closest phenotype for comparison. [3]iPSYCH was not included in Trubetskoy et al, 2022 and was therefore reported under "PGC excluding iPSYCH".

File Name: Supplementary Data 9
Description: Mental- and cardiometabolic disorders: International Classification of Disease (ICD) codes.
