# Supplementary material for: Quantifying the relative importance of genetics and environment on the comorbidity between mental and cardiometabolic disorders using 17 million Scandinavians
Source: Nat Commun. 2024 Jun 13;15:5064. doi: 10.1038/s41467-024-49507-3 (PMC11176385; doi:10.1038/s41467-024-49507-3)
Supplement: Supplementary file 5 — Reporting Summary [file 41467_2024_49507_MOESM5_ESM.pdf]

nature portfolio

Corresponding author(s): Joeri Meijsen
Alfonso Buil

Last updated by author(s): 02-05-2024

Reporting Summary

Nature Portfolio wishes to improve the reproducibility of the work that we publish. This form provides structure for consistency and transparency in reporting. For further information on Nature Portfolio policies, see our Editorial Policies and the Editorial Policy Checklist.

Statistics

For all statistical analyses, confirm that the following items are present in the figure legend, table legend, main text, or Methods section.

n/a	Confirmed	
☐	☒	The exact sample size (*n*) for each experimental group/condition, given as a discrete number and unit of measurement
☒	☐	A statement on whether measurements were taken from distinct samples or whether the same sample was measured repeatedly
☐	☒	The statistical test(s) used AND whether they are one- or two-sided<br>*Only common tests should be described solely by name; describe more complex techniques in the Methods section.*
☒	☐	A description of all covariates tested
☐	☒	A description of any assumptions or corrections, such as tests of normality and adjustment for multiple comparisons
☐	☒	A full description of the statistical parameters including central tendency (e.g. means) or other basic estimates (e.g. regression coefficient) AND variation (e.g. standard deviation) or associated estimates of uncertainty (e.g. confidence intervals)
☐	☒	For null hypothesis testing, the test statistic (e.g. *F*, *t*, *r*) with confidence intervals, effect sizes, degrees of freedom and *P* value noted<br>*Give P values as exact values whenever suitable.*
☒	☐	For Bayesian analysis, information on the choice of priors and Markov chain Monte Carlo settings
☐	☒	For hierarchical and complex designs, identification of the appropriate level for tests and full reporting of outcomes
☒	☐	Estimates of effect sizes (e.g. Cohen's *d*, Pearson's *r*), indicating how they were calculated

Our web collection on statistics for biologists contains articles on many of the points above.

Software and code

Policy information about availability of computer code

Data collection	No software was used for data collection
Data analysis	Danish register data was stored on a PostgreSQL 13.3 database server information was extracted using the psql 16.2 database client. All register based analyses were done in R v4.2.1 using the cmprsk v2.2 package. All analyses performed on genotype data (GWAS summary statistics) were performed using linkage-disequilibrium score regression (LDSC) version 1.0.1. GWAS summary statistics were clearned using the cleansumstats pipeline (reference 56). Mathematical formulas to calculate h2 and rg were taken from reference 20.

For manuscripts utilizing custom algorithms or software that are central to the research but not yet described in published literature, software must be made available to editors and reviewers. We strongly encourage code deposition in a community repository (e.g. GitHub). See the Nature Portfolio guidelines for submitting code & software for further information.

Data

Policy information about availability of data

All manuscripts must include a data availability statement. This statement should provide the following information, where applicable:

- Accession codes, unique identifiers, or web links for publicly available datasets
- A description of any restrictions on data availability
- For clinical datasets or third party data, please ensure that the statement adheres to our policy

The use of Danish data was approved by the Danish Health Data Authority (project no. FSEID-00003339) and the Danish Data Protection Agency. The use of Swedish

data was approved by the regional ethics review board in Stockholm, Sweden with DNR 2012/1814-31/4. By Danish and Swedish law, informed consent is not required for register-based studies. The raw Danish and Swedish register data are protected and are not available due to data privacy laws. Only Danish research environments are granted authorization to Danish data. Foreign researchers can, however, get access to data. Further information on data access can be found at https://www.dst.dk/en/TilSalg/Forskningsservice or by contacting the senior corresponding authors. Data from Swedish registers are not available for sharing due to policies and regulations in Sweden. Swedish register data are available to all researchers through applications at Statistics Sweden (SCB, https://www.scb.se/en/) and The National Board of Health and Welfare (Socialstyrelsen, https://www.socialstyrelsen.se/) The data generated in this study are provided in the Supplementary Information/Source Data file. GWAS summary statistics including stroke (subtypes), CAD, aneurysms and HF were obtained through multiple public repositories. GWAS summary statistics containing participants of the VA Million Veterans Program (e.g., T2D, venous thromboembolism, and peripheral artery disease) were provided after approval was granted by the National Institute of Health (project #26508). GWAS summary statistics for ADHD, AN, ASD, BD, and MDD excluding iPSYCH participants (except SCZ which does not contain iPSYCH samples) were kindly provided through their respective PGC consortium. iPSYCH only GWAS summary statistics for Mental disorders were downloaded from internal iPSYCH servers and are available on request.

Research involving human participants, their data, or biological material

Policy information about studies with human participants or human data. See also policy information about sex, gender (identity/presentation), and sexual orientation and race, ethnicity and racism.

Reporting on sex and gender

The mentioning of the term "sex" occurs only once in this manuscript and relates, for the fast majority of individuals born in Denmark and Sweden, to biological attributes reported at birth. We are concious about the fact that for a small number of individuals this variable should be refered to as "Gender" as individuals can request a new social security numbers with a new name and change their reported "sex" to their prefered "gender".

In this study we did not consider sex and no sex specific analyses (e.g., male or female only) analyses were performed. We consider this paper a first pass publication to justify our method. We have already explored and implemented sex stratified analyses in upcoming publications.

Reporting on race, ethnicity, or other socially relevant groupings

In this study we did not use any metric, to either seperate or remove individuals related to: "race", ethnicity, ancestry, or other socially relevant groupings as to the best of our knowledge no such information is recorded in any of the registers in both Denmark and Sweden.

Population characteristics

Analyses were performed using 2 cohorts: 1.) the full Danish (n=7,797,622, Age=51.5, female=49.6%) or Swedish (n=13,222,453, Age=53.5, %female=49.3) register using medical data up to 2016 and 2.) individuals born between 1981 and 2005 using diagnoses made no later than 2012 (Denmark: n=1,560,901, Age=18.8, female=48.7; Sweden: n=2,566,100, Age=19.5, %female=48.6). Analysis were performed per birth year to account for age and birth year related cohort effects.

Recruitment

In this study individuals were not recruited. Here we made use of register based data recorded by the governments of Denmark and Sweden which are open for scientific use. By their respective nationals laws, informed consent is not required for register-based studies.

Ethics oversight

The use of Danish data was approved by the Danish Health Data Authority (project no. FSEID-00003339) and the Danish Data Protection Agency. The use of Swedish data was approved by the regional ethics review board in Stockholm, Sweden with DNR 2012/1814-31/4.

Note that full information on the approval of the study protocol must also be provided in the manuscript.

Field-specific reporting

Please select the one below that is the best fit for your research. If you are not sure, read the appropriate sections before making your selection.

☒ Life sciences ☐ Behavioural & social sciences ☐ Ecological, evolutionary & environmental sciences

For a reference copy of the document with all sections, see nature.com/documents/nr-reporting-summary-flat.pdf

Life sciences study design

All studies must disclose on these points even when the disclosure is negative.

Sample size

No predetermined sample size calculation was performed. Our data included the full population of both Denmark and Sweden that have been hospitalised between 1972 and 2016.

Data exclusions

No data collection was performed. This study uses observational data from administrative register of two countries. Individuals were removed if the individual was born outside of Denmark and/or Sweden OR the individual had an MDD, BIP, or SCZ diagnosis made before age 10 OR when a specific diagnosis was made after age 100.

Replication

Initial analysis was performed in Denmark and replication analysis was performed in Sweden. All heritability estimates derived using Danish data replicated in Sweden (21/21) and all but 1 genetic correlations calculated using Danish data replicated using Swedish data (1/90), after correcting for multiple testing. All analyses performed are reported.

Randomization

Since this is an observational study, no randomization was performed. In the comparison between general population estimates (by means of a cumulative incidence) and individuals with at least one affected family member, we matched individuals according to year of birth.

Blinding | Since this is an observational study, there were no experiments warranting blinding, and statistical analyses were exploratory and therefore not blinded.

Reporting for specific materials, systems and methods

We require information from authors about some types of materials, experimental systems and methods used in many studies. Here, indicate whether each material, system or method listed is relevant to your study. If you are not sure if a list item applies to your research, read the appropriate section before selecting a response.

Materials & experimental systems

n/a	Involved in the study
☒	☐ Antibodies
☒	☐ Eukaryotic cell lines
☒	☐ Palaeontology and archaeology
☒	☐ Animals and other organisms
☒	☐ Clinical data
☒	☐ Dual use research of concern
☒	☐ Plants

Methods

n/a	Involved in the study
☒	☐ ChIP-seq
☒	☐ Flow cytometry
☒	☐ MRI-based neuroimaging

Plants

Seed stocks | N/A

Novel plant genotypes | N/A

Authentication | N/A
